# Contribution of Ascorbate and Glutathione in Endobacteria *Bacillus subtilis*-Mediated Drought Tolerance in Two *Triticum aestivum* L. Genotypes Contrasting in Drought Sensitivity

**DOI:** 10.3390/plants10122557

**Published:** 2021-11-23

**Authors:** Dilara Maslennikova, Oksana Lastochkina

**Affiliations:** Institute of Biochemistry and Genetics, Subdivision of the Ufa Federal Research Centre of the Russian Academy of Sciences, 450054 Ufa, Russia; dishaoil@mail.ru

**Keywords:** endophytic bacteria, *Bacillus subtilis*, *Triticum aestivum* L., drought, tolerance, hydrogen peroxide, malondialdehyde, ascorbate, glutathione

## Abstract

We evaluated the effect of endobacteria *Bacillus subtilis* (strain 10–4) as a co-inoculant for promoting plant growth and redox metabolism in two contrasting genotypes of *Triticum aestivum* L. (wheat): Ekada70 (drought tolerant (DT)) and Salavat Yulaev (drought susceptible (DS)) in early stages of adaptation to drought (12% PEG–6000). Results revealed that drought reduced growth and dramatically augmented oxidative stress markers, i.e., hydrogen peroxide (H_2_O_2_) and lipid peroxidation (MDA). Furthermore, the depletion of ascorbate (AsA) and glutathione (GSH), accompanied by a significant activation of ascorbate peroxidase (APX) and glutathione reductase (GR), in both stressed wheat cultivars (which was more pronounced in DS genotype) was found. *B. subtilis* had a protective effect on growth and antioxidant status, wherein the stabilization of AsA and GSH levels was revealed. This was accompanied by a decrease of drought-caused APX and GR activation in DS plants, while in DT plants additional antioxidant accumulation and GR activation were observed. H_2_O_2_ and MDA were considerably reduced in both drought-stressed wheat genotypes because of the application of *B. subtilis*. Thus, the findings suggest the key roles in *B. subtilis*-mediated drought tolerance in DS cv. Salavat Yulaev and DT cv. Ekada70 played are AsA and GSH, respectively; which, in both cases, resulted in reduced cell oxidative damage and improved growth in seedlings under drought.

## 1. Introduction

Drought is one of the most dominant abiotic stresses hindering growth and yield (by up to 80%) in crop plants and affects about 64% of the world’s agricultural lands [1,2,3,4]. Drought conditions impair the general metabolism of plants at physiological, biochemical, and molecular levels, leading to growth inhibition. Drought causes an overproduction of reactive oxygen species (ROS), which leads to a change in cellular redox homeostasis, inactivation of membrane-bound proteins, decreased membrane fluidity, DNA damage, and inhibition of protein synthesis and enzymatic activity [1,3,5,6]. In nature, plants are equipped with various defense systems to counter external threats, including enzymatic and non-enzymatic antioxidants [5,6]. However, their protective effects are insufficient under severe drought conditions, resulting in significant yield losses in all types of crops, including such strategically important food cereals as bread wheat (*Triticum aestivum* L.). This creates a natural demand for new approaches that enhance natural plant defense systems. The negative effects of abiotic stresses on plants, drought in particular, can be successfully overcome by plant growth promoting bacteria (PGPB), which are natural inhabitants of the rhizosphere soil [7,8,9,10].

Endophytic PGPB, particularly *Bacillus subtilis*, are a cheap eco-friendly biological strategy for improving plant growth/productivity under drought conditions. They function by activating the natural defense mechanisms of host plants without causing a negative impact on them, the environment, or human health [7,8,11]. The ability of *B. subtilis* to improve wheat growth and tolerance under various abiotic stresses has been reported in many studies [7,10,12,13]. It is presumed that *Bacillus* exerts a protective effect on stressed plants by colonizing external (rhizobacteria) and/or internal (endobacteria) host tissue, positively modulating plant metabolism, improving the absorption of water and nutrients by producing various bioactive compounds, and inducing multiple plant defense reactions [10,11,12,14]. However, many aspects of the interaction between endophytic *B. subtilis* and plants (wheat) under drought stress remain unclear and require further detailed investigation. Additionally, the effectiveness of the same *B. subtilis* strain can vary depending on plant genotype, environmental stresses, and many other factors [4].

A crucial step in improving drought tolerance in plants is detoxification of stress-caused ROS by utilizing an antioxidant defense mechanism that involves a number of antioxidants, both enzymatic (superoxide dismutase, peroxidase, catalase, ascorbate peroxidase (APX), and glutathione reductase (GR)) and non-enzymatic (ascorbate (AsA), glutathione (GSH), cysteine, proline, flavonoids, carotenoids, and tocopherol) [5,6,15]. There is now substantial evidence that different cereals inoculated with PGPBs can survive under abiotic stress by reducing stress-caused oxidative damages as judged by lipid peroxidation and electrolytes leakages [7,10,14,16,17,18]. It has also been demonstrated that *Bacillus* leads to changes in the balance of ROS and the activity of antioxidant enzymes in wheat under drought [8,10], which plays an important role in the manifestation of pre-adaptive and anti-stress action of this bacterium on plants [8,10,19]. Among antioxidants, AsA and GSH are the most important in plants. Both molecules scavenge ROS and are components of the pivotal antioxidant Foyer–Halliwell–Asada pathway or AsA–GSH cycle [5,6]. The studies shows that wheat plant responses to drought-induced oxidative stress involve AsA–GSH cycle metabolism [6,15]. Despite the accumulated data on the ability of *B. subtilis* to reduce drought-caused oxidative damages in plants, it is still not clear how exactly they can influence the AsA–GSH metabolism, and what their contribution is to the bacterial-mediated drought tolerance of different wheat genotypes.

The aim of the present study was to assess the influences of endobacteria *B. subtilis* 10–4 on the growth, cellular oxidative status (MDA, H_2_O_2_), and the components of the AsA–GSH complex (AsA, GSH, GSSG, APX, GR) in two wheat (*T. aestivum* L.) genotypes with contrasting drought sensitivity and grown under drought (12% PEG–6000) in early stages of ontogenesis.

## 2. Results

### 2.1. Growth Attributes

Analysis carried out in B. subtilis-treated wheat seeds three days post germination in the presence of 12% PEG–6000 (drought simulation) revealed that drought significantly decreased the germination rate by 57% (under control 97%) and 39% (under control 96%) for DT cv. Ekada70 and DS cv. Salavat Yulaev, respectively (Table 1). Seed treatment with *B. subtilis* 10–4 improved the percentage of seed germination up to 74% for DT genotype, while for DS genotype an inhibitory effect (the percentage of germination was only 27%) was observed. However, under normal growth conditions (0% PEG) *B. subtilis* 10–4 contributed to some increase in the seed germination of both wheat genotypes (Table 1).

Further analysis was carried out in early wheat seedlings hydroponically grown during four days under normal conditions and then transferred to 12%PEG–6000 (drought) for 7 h. It was observed that exposure to drought for 7 h led to inhibition in the growth of early wheat seedlings in both genotypes (Table 1). Drought reduced the length of shoots and roots by 15–20% in DT cv. Ekada70 (Table 1), and by 10–25% in DS cv. Salavat Yulaev (Table 1). Pre-sowing seed treatment with *B. subtilis* 10–4 mitigated the negative impact of drought on the growth of early wheat seedlings of both genotypes. Under normal (non-stressed) growth conditions, *B. subtilis* 10–4 also demonstrated growth-promoting activity on both genotypes with better effect on DT cv. Ekada70.

### 2.2. H_2_O_2_ and Lipid Peroxidation (MDA)

**Figure 1:**
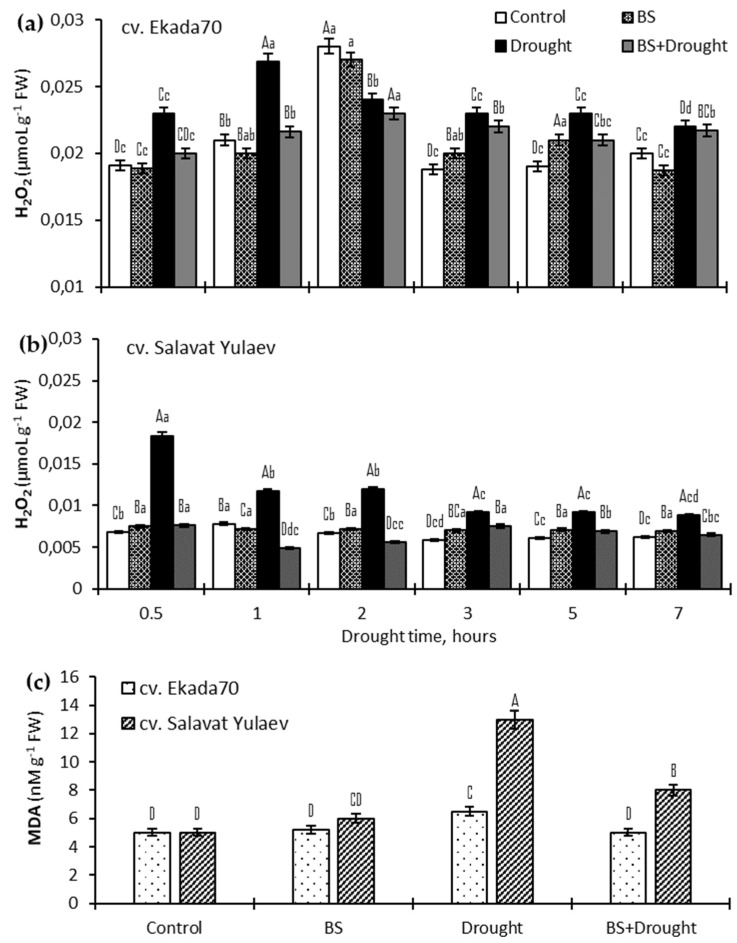
Influence of endobacteria *B. subtilis* 10–4 (BS) pre-treatment on hydrogen peroxide (H_2_O_2_) (**a**,**b**) in roots and lipid peroxidation (MDA) (**c**) in four-days old *T. aestivum* L. (wheat) seedlings of drought tolerant (DT) cv. Ekada 70 and drought sensitive (DS) cv. Salavat Yulaev under normal and drought (12% PEG–6000) conditions. MDA was estimated in wheat seedlings exposed to drought for 7 h (**c**). The bars indicate the mean values of three repetitions ± SEM. Different lowercase letters on top of the columns indicate that means for each treatment at different time points are different at *p* < 0.05. Different capital letters on top of the columns indicate that means for the same time point of various treatments are different at *p* < 0.05.

It was observed that drought led to significant hydrogen peroxide (H_2_O_2_) generation in root tissues of both wheat genotypes (Figure 1). In the roots of DT cv. Ekada70, H_2_O_2_ levels increased by about 130% with a peak at 1 h of drought exposure (Figure 1a). In the roots of DS cv. Salavat Yulaev, a transit (almost by 300%) increase in H_2_O_2_ was recorded at 0.5 h of stress (Figure 1b). Pre-treatment with *B. subtilis* 10–4 contributed to the maintenance of H_2_O_2_ in both genotypes at levels similar to control values (Figure 1a,b). It should also be noted that in non-stressed plants the initial H_2_O_2_ concentration was higher in DT cv. Ekada70 in comparison with plants of DS cv. Salavat Yulaev in the same growth conditions (Figure 1a,b).

A remarkable increase (by 260%) in lipid peroxidation (MDA) was observed after 7 h of drought exposure in DS cv. Salavat Yulaev (Figure 1c). In contrast, a corresponding increase of MDA in DT cv. Ekada70 seedlings was only 130%. Conversely, MDA was significantly reduced in the stressed wheat seedlings pre-treated with *B. subtilis* 10–4. This effect was more pronounced (almost reaching control levels) for seedlings of DT genotype (Figure 1c), but in seedlings of DS genotype MDA declined by 62%. Additionally, the results summarized in Figure 1c show that under normal growth conditions the concentration of MDA did not change in bacterial-inoculated DT genotype, but a slight increase (by 115%) in DS genotype in comparison with non-inoculated control wheat seedlings was observed under drought conditions.

### 2.3. Ascorbic Acid (AsA) and Glutathione (GSH)

**Figure 2:**
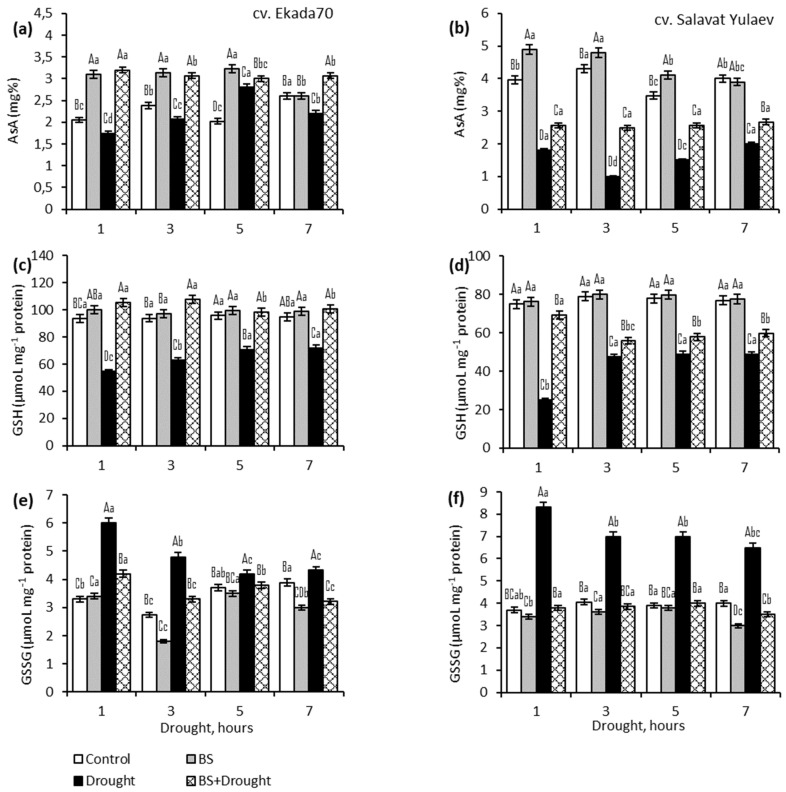
Effect of *B. subtilis* 10–4 (BS) pre-treatment on the content of some non-enzymatic antioxidants (ascorbate—AsA (**a**,**b**), reduced glutathione—GSH (**c**,**d**), and oxidized glutathione—GSSG (**e**,**f**)) in two *Triticum aestivum L*. early seedlings (four-days old) differing in drought sensitivity (drought tolerant (DT) cv. Ekada70 (**a**,**c**,**e**); drought sensitive (DS) cv. Salavat Yulaev (**b**,**d**,**f**)) grown under normal (control) and drought conditions. The bars indicate the mean values of three repetitions ± SEM. Different lowercase letters on top of the columns indicate that means for each treatment at different time points are different at *p* < 0.05. Different capital letters on top of the columns indicate that means for the same time point of various treatments are different at *p* < 0.05.

This study demonstrated differing ASA and GSH content in young DT cv. Ekada70 vs. DS cv. Salavat Yulaev wheat seedlings (Figure 2). So, under normal growth conditions, seedlings of cv. Ekada70 were characterized by an insignificant, but reliably higher (by 124%) GSH content (Figure 2a) compared to seedlings of cv. Salavat Yulaev (Figure 2b). At the same time, the seedlings of DS cv. Salavat Yulaev contained significantly more AsA (160%). Pre-treatment with *B. subtilis* 10–4 led to additional AsA accumulation in seedlings of both cultivars, with the greatest accumulation throughout the experiment observed in DT cv. Ekada70 (approximately 150% of control). Whereas, in the cv. Salavat Yulaev it was in the range of 123–125% relative to the control level. In *Bacillus*-inoculated plants the GSH did not change significantly, although a slight accumulation was observed. Additionally, the level of oxidized GSH (GSSG) in plants of both cultivars pre-inoculated with *B. subtilis* 10–4 remained unchanged (Figure 2e,f). This indicates the absence of a negative effect of bacterial treatment on the physiological state of plants.

It was found that the plants of DS cv. Salavat Yulaev were more susceptible to the effects of drought, as evidenced by a sharp two-fold drop in AsA throughout the entire experiment and an almost three-fold decrease in GSH (Figure 2d), which correlates with the same level of GSSG (Figure 2b). Evaluation of the AsA and GSH contents under drought conditions in plants of cv. Ekada70 demonstrated drought tolerance—the decrease in the AsA content was about 20% relative to the control level (Figure 2a). While the glutathione system reacts to stress brighter, as evidenced by an average 1.5 times dropping in GSH during the total duration of stress (Figure 2c), and by 2 times increasing of GSSG by 1 h of stress exposure and a noticeable decrease by 5 h of stress exposure (Figure 2e).

Under drought conditions, the plants of DS cv. Salavat Yulaev were found to have a sharp decrease of AsA (by 200%) (Figure 2b) and GSH (by 300%) (Figure 2d) throughout the entire experiment. In plants of DT cv. Ekada70, drought led to a smaller decrease of AsA (by 20%) (Figure 2a) and GSH (by 150%) (Figure 2c) throughout the entire period of stress in comparison with DS genotype. Moreover, it was found that drought exposure for 1 h increased GSSG in both DT (by 176%) (Figure 2e) and DS (by 224%) (Figure 2f) wheat seedlings. However, during further drought exposure (from 3 to 7 h) GSSG were varied between 110–170% and 163–174% for DT and DS genotypes, respectively.

*B. subtilis* 10–4 did not prevent the stress-mediated decrease of AsA in plants of DS cv. Salavat Yulaev (Figure 2b), but rather contributed to the maintenance of its increased level (by 130–250%) in comparison with control (non-bacterized) and stressed plants. At the same time, in these plants GSH was higher than the values of stressed plants (Figure 2d), but lower than the control. In parallel, a restoration of GSSG at the level of control values was observed (Figure 2f). In plants of DT cv. Ekada70, pre-treatment with *B. subtilis* 10–4 stabilized GSH and AsA at the level of control under drought (Figure 2a,c,e).

### 2.4. The Activity of Ascorbate Peroxidase (APX) and Glutathione Reductase (GR)

**Figure 3:**
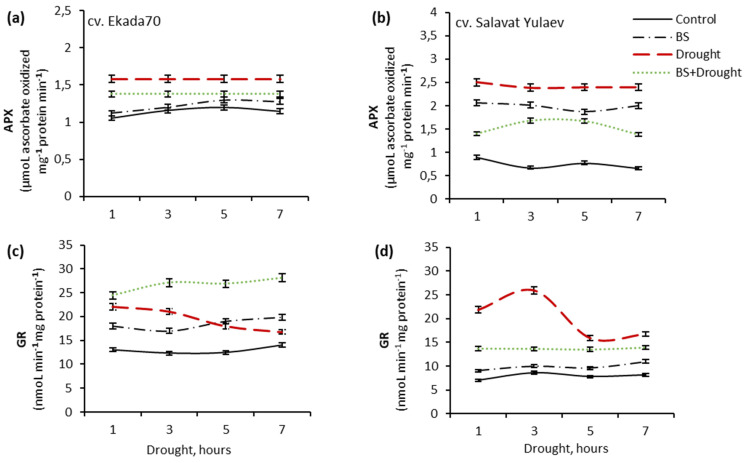
Influence of *B. subtilis* 10–4 (BS) pre-treatment on the activity of ascorbate peroxidase (APX) (**a**,**b**) and glutathione reductase (GR) (**c**,**d**) in two *T. aestivum* L. seedlings (four-days old) differing in drought sensitivity (drought tolerant (DT) cv. Ekada 70 (**a**,**c**); drought sensitive (DS) cv. Salavat Yulaev (**b**,**d**)) grown under normal (control) and drought (12% PEG–6000) conditions. The bars indicate the mean values of three repetitions ± SEM.

The results presented in Figure 3 indicate a pronounced activation of APX and GR in both wheat genotypes (Figure 3), but the response amplitude was higher in seedlings of DS cv. Salavat Yulaev (Figure 3b,d), where drought caused a more than 300% and 200% increase in APX and GR activities, respectively. In contrast, in seedings of cv. Ekada70 the activities of APX and GR increased approximately by 150% and 120–170%, respectively (Figure 3c). Furthermore, the reduction of GR activity in both genotypes after 3 h of stress exposure was observed (Figure 3c,d).

Pre-treatment with *B. subtilis* 10–4 under drought led to a decrease in APX (by 142–173%) and GR (by 120–192%) activities in plants of DS cv. Salavat Yulaev (Figure 3b,d). Whereas, in plants of DT cv. Ekada70, a smaller decrease (by 115–120% under control) in APX activity and an additional activation of GR (by 125–130%) were observed (Figure 3a,c). Bacterial treatment significantly reduced (by 114–140%) stress-caused APX activity in seedlings of cv. Ekada70 (Figure 3a) and resulted in additional GR activation in them, reaching a 200% increase by 7 h of stress exposure (Figure 3c). It should be noted, under normal growth conditions *B. subtilis* 10–4 resulted in different activations of APX and GR in both wheat genotypes: by 260% and 140%, respectively, in plants of cv. Salavat Yulaev (Figure 3b,d); by 115% and 150%, respectively, in plants of cv. Ekada70 (Figure 3a,c).

## 3. Discussion

Drought is a major environmental stress that limits plant growth and inhibits many other cellular developmental functions with the effect of reducing crop productivity worldwide [1,2,3,4,5]. In our work, we used two wheat genotypes, which differ significantly in the degree of drought sensitivity (drought-tolerant (DT) cv. Ekada70 and drought-susceptible (DS) cv. Salavat Yulaev)). It thus comes as no surprise that drought stress led to significant inhibition of seedling growth in both cultivars. The application of *B. subtilis* 10–4 had a growth-stimulating effect under normal conditions and protective effect under drought conditions on both DS and DT early wheat seedlings. These results are consistent with other studies in which a marked increase in wheat plant growth and tolerance was observed in response to PGPB treatment [10,16]. However, our results also suggest wheat genotype (cultivar)-specific physiological action of the same strain *B. subtilis* 10–4 on seed germination showing the best positive effect on DT cv. Ekada70.

It is of great interest to compare two wheat genotypes contrasting in drought sensitivity according to the state of the components of the antioxidant system, as this system is one of the most critical mechanisms that confer resistance/tolerance to stress in plants. The intensity of oxidative processes in the cell can be judged by the content of the end product of lipid peroxidation—MDA, as well as hydrogen peroxide (H_2_O_2_) and superoxide (O_2_^•−^), which are normally present in tissues in rather low concentrations. In many studies in stressed plants, the contents of MDA and H_2_O_2_ notably declined thanks to PGPB treatment [10,14,17,18,19]. For example, non-bacterized and drought exposed okra plants generated ROS to a more lethal level than in bacterized plants [19]. The findings indicate that PGPB alters the rate of metabolism by activating a strong ROS scavenging system in stressed plants. These may accelerate the restoration of membrane stability and protect against photodamage during re-watering in bacterial treated plants [19]. Our results also showed *B. subtilis* decreases lipid peroxidation and H_2_O_2_ in wheat of both genotypes under drought (Figure 1). Interestingly, the bacterial introduction (before stress) was able to independently activate some antioxidant enzymes. The ability of *B. subtilis* under normal growth conditions to slightly increase H_2_O_2_ in roots of wheat seedlings (Figure 1) may play an important role in biochemical processes associated with biosynthesis of suberin and lignin [20]. This can help to strengthen the cell walls and subsequently ensure effective neutralization of the excess (damaging) stress-induced ROS increase, as well as prevent disruption of the integrity of cells’ membrane structures and changes in their permeability under stress conditions. In favor of this, in our previous study, we demonstrated *B. subtilis* 10–4 intensified lignin deposition in roots of bean plants under normal conditions and exerted a protective effect under salinity decreasing oxidative damages [21]. Perhaps *B. subtilis* is perceived by plants as an agent that causes initial intracellular changes in the antioxidant system which, however, do not lead to seedling damage (Figure 1). Thus, a low ROS production may be important for the pre-adaptive action of *Bacillus* on plants to subsequent stressful situations since ROS are known to act as signaling molecules in the initiation of a cascade of defense reactions in plants [5,6]. However, a sharp stress-induced increase in ROS can lead to serious destructive consequences [1,5,6]. There is now substantial evidence that different plants inoculated with various PGPBs can survive under abiotic stress-caused oxidative damages by acting on antioxidant enzymes [10,19,22,23]. The increased activity of SOD, POD, CAT, APX, and GR in PGPB-inoculated plants has been reported as one of the main mechanisms of microbe-induced plant protection against drought [7,10,18,22].

The key role in stabilizing the redox status is assigned to non-enzymatic antioxidants AsA and GSH, which are involved in the direct neutralization of an excess of ROS, and are also the most important participants in the AsA–GSH cycle (or Halliwell–Assad pathway) [5,6]. In this cycle, APX catalyzes the reduction of H_2_O_2_ into the water with reduced AsA acting as an electron donor. Dehydroascorbate reductase (DHAR) utilizes the electrons provided by GSH to reduce dehydroascorbic acid (DHA), which is previously produced from monodehydroascorbate (MDHA). Simultaneously, reduced GSH is oxidized into GSSG by DHAR, and GSSG is then reduced into GSH, catalyzed by GR. The AsA–GSH cycle can maintain an appropriate oxidative and reductive environment in cells through regulating three interdependent redox couples: AsA/DHA, GSH/GSSG, and NAD(P)H/NAD+(P) [6]. According to the literature data, the accumulation of AsA and GSH in drought-stressed okra plants inoculated with *B. subtilis* indicates activation of the AsA–GSH cycle, promoting active trapping of drought-generated H_2_O_2_ [19]. *B. amyloliquefaciens* 5113 had a protective effect on wheat plants during drought by regulating the activity of the ascorbate-peroxidase *APX1* gene and enzymes of the AsA–GSH complex due to the high intracellular content of AsA and GSH, which provides a high buffer redox capacity of cells and serves as a key player in the antioxidant defense system [24]. Interestingly, under normal conditions, *B. velezensis* 5113 had practically no effect on the AsA–GSH cycle in wheat but significantly reduced APX activity under heat stress. However, under drought and cold stress conditions, there was no significant decrease was found [25]. In addition, it was reported that drought led to an increase in the activity of such stress-related genes as *APX1*, *SAMS1*, and *HSP17.8* in wheat leaves and increased the activity of the AsA–GSH cycle’ enzymes. While in the same bacterial-treated plants, the number of transcripts of these genes decreased, which indicates an improvement in the state of homeostatic mechanisms due to bacterial priming [26]. Obviously, *Bacillus* strains can reduce the degree of oxidative damage in different ways with the involvement of various biochemical pathways, which may depend on many factors, including the characteristics of the strain itself, the type of plant, the type of stress, and its intensity. Our results revealed that DT cv. Ekada70 has more GSH and increased GR activity, and this possibly determines a slight drop in AsA and GSH under stress (Figure 2). Most likely this determines drought tolerance of cv. Ekada70 in comparison with cv. Salavat Yulaev, which is declared drought-susceptible. This is also supported by obtained data on the pronounced drop in AsA and GSH levels in drought-stressed cv. Salavat Yulaev. Drought led to the strongest APX and GR activation, but the response amplitude was higher in DS wheat plants than in DT ones. The result of these enzymes’ work was reflected in H_2_O_2_, MDA, AsA, and GSH, as well as the growth rates of these plants. Assessment of AsA and GSH in bacterized plants under drought showed that their level was higher compared to non-bacterized plants under the same conditions. In plants of the DS cv. Salavat Yulaev, *B. subtilis* promotes an increase in the level of antioxidants and practically restores the level of GSSG. We associate this effect with the fact that *B. subtilis* 10–4 significantly reduced the activity of APX and GR, and these antioxidants were depleted to a much lesser extent. It should be noted that in these plants, under normal growth conditions, *B. subtilis* 10–4 significantly increased APX activity, while GR was slightly activated. In seedlings of cv. Ekada70, the bacterial treatment led to an almost complete restoration of the AsA and GSH contents. We suggest this effect with the fact that bacteria under normal growth conditions led to a small but reliable additional AsA accumulation and its activation by GR, which, in our opinion, provides rapid complete recovery of GSH and decrease in GSSG levels. Thus, the results indicate the key role of AsA and GSH in the formation of *B. subtilis*-induced drought tolerance of wheat genotypes differing in drought sensitivity.

## 4. Materials and Methods

### 4.1. Bacterial Strain and Inoculum Preparation

The endophytic bacterium *B. subtilis* (strain 10–4) was isolated from the arable soils at the Bashkir Research Institute of Agriculture of the Ufa Federal Research Center at the Russian Academy of Sciences (BRIA UFRC RAS) (Ufa, Russia), identified using 16S rRNA, characterized [14,18] and deposited in the Russian National Collection of Industrial Microorganisms (VKPM) (registration number B-12988). *B. subtilis* 10–4 cells were cultured in LB solid agar medium at 37 °C for 24 h [27]. To obtain inoculum of *B. subtilis* 10–4, the suspension was prepared containing 10^8^ CFU mL^−1^ of bacteria according to 0.5 McFarland turbidity standard and monitored at the optical density 600 nm (OD600) (SmartSpecTM Plus spectrophotometer, Bio–Rad, USA), and then diluted down to 10^5^ CFU mL^−1^ using sterile water.

### 4.2. Plant Materials and Growth Conditions

Wheat seeds (*Triticum aestivum* L., BBAADD 2n = 42) of drought-susceptible (DS) cv. Salavat Yulaev (SY,) and drought-tolerant (DT) cv. Ekada70 were obtained from the Chishminsky Breeding Station, UFRC RAS (Russia). The seeds were sterilized in 97% ethyl alcohol for 1 min, then washed with sterile tap water for 2–3 min (until the smell of alcohol disappeared). Thereafter, the seeds were immersed into solutions of *B. subtilis* 10–4 (10^5^ CFU mL^−1^) or sterile water (control) for 1 h. The treated seeds were hydroponically grown (on filter paper moistened with tap water) under a 16/8 h light/dark photoperiod regime under (200 μmoL m^−2^ s^−1^) at 22–24 °C for four days. Thereafter, the seedlings were transferred to glasses with distilled water (control) or 12% PEG–6000 (drought) and grew further at the same conditions. Plant samples (root, shoots, or whole seedlings) were taken after 0.5, 1, 2, 3, 5, 7 h (depending on purposes) to assess physio-biochemical attributes.

### 4.3. Growth Parameters

To assess wheat seed germination percentage, the seeds pre-treated with *B. subtilis* 10–4 and non-treated (control) germinated in Petri dishes in the presence of 12% PEG–6000 (drought) and distilled water (normal growth condition) for three days. The length of wheat seedlings’ roots and shoots were measured with a ruler in four-days old hydroponically grown seedlings exposed to drought for 7 h. Fresh weight (FW) of roots and shoots were recorded on the same day. To measure the dry weight (DW), samples were placed in an oven at 70 °C, and after 48 h measurements were conducted [28].

### 4.4. Malondialdehyde (MDA) and Hydrogen Peroxide (H_2_O_2_) Contents

MDA and H_2_O_2_ were measured as biomarkers of lipid peroxidation and oxidative stress. To determine MDA [28], wheat seedlings were ground in distilled water and then homogenized in 20% trichloroacetic acid. The homogeneous samples then were centrifuged (10,000× *g*, 10 min). Then the supernatant was mixed with 0.5% thiobarbituric acid prepared in 20% trichloroacetic acid and was kept in a boiling water bath (100 °C, 30 min), and quickly cooled. Absorbance was spectrophotometrically (SmartSpecTM Plus, Bio–Rad, CA, USA) measured at 532 nm and 600 nm. The MDA was expressed as nmoL g^–1^ FW.

To determine H_2_O_2_ [29], samples of plant material (roots) homogenized (1:5 weight/volume) in 0.05M sodium phosphate buffer (FB), pH 6.2. The supernatant was separated by centrifugation (Eppendorf^®^ Microcentrifuge 5415 R, Humburg, Germany) at 15,000× *g* for 15 min. The concentration of H_2_O_2_ in the supernatant was spectrophotometrically (SmartSpecTM Plus, Bio–Rad, Hercules, CA, USA) determine using xylenol orange in the presence of Fe^2+^ at 560 nm. The H_2_O_2_ was expressed as µmoL g^–1^ FW.

### 4.5. Measurement of Non-Enzymatic Antioxidants

#### 4.5.1. Total of Reduced Glutathione (GSH) and Oxidized Glutathione (GSSG) Content

Content of GSH and GSSG forms of glutathione from the same plant sample were determined using a spectrofluorimetric method based on the formation of a fluorescent product o-phthalaldehyde (Sigma, Australia) dependent on pH of the medium. Plant samples (0.5 g) were homogenized in 4 mL of the mixture containing 0.1 M potassium phosphate buffer (pH 8.0) and 25% metaphosphoric acid at a ratio of 3.75:1 (by volume) according to Hissin and Hilf [30]. Homogenate was centrifuged for 10 min at 8000× *g*, and then supernatant was repeatedly centrifuged for 5 min at 13,000× *g*. GSH and GSSG in the obtained supernatant were quantified using reagents described in detail by Maslennikova et al. [15]. In order to determine the content of GSH and GSSG, we monitored the kinetics of the fluorescence strength of the formed complexes at a temperature of 25 °C using an EnSpire Model 2300 Multilabel Microplate Reader (PerkinElmer, Boston, MA, USA) at 420 nm (excitation wavelength of 350 nm). The content of GSH and GSSG was expressed in µmoL mg^−1^ protein.

#### 4.5.2. Ascorbate (AsA) content

AsA content was determined using the titration method [31]. Plant samples (10 g) were ground in a porcelain mortar, extracted with 10 mL of distilled water, stirred, and filtered through a paper filter. An amount of 20 mL of filtrate was taken into a conical flask and 1 mL of 2% HCl, 0.5 mL of 1% KI, and 2 mL of 0.5% starch were added, stirred, and titrated with 0.001 moL L^−1^ KIO_3_ until stable blue color staining. The ascorbate content was expressed in mg % FW.

### 4.6. Measurement of Enzymatic Antioxidants

#### 4.6.1. Ascorbate Peroxidase (APX) Activity

Ascorbate peroxidase (APX, EC 1.11.1.11) activity was estimated by monitoring ascorbate oxidation at 290 nm [32]. The reaction mixture (2.93 mL) consisted of 50 mM phosphate buffer (pH 7.0), (0.03 mL) 17 mM ASC, (0.03 mL) EDTA, 0.01 mL extract. The reaction was started by the addition of 0.03 mL 0.06% H_2_O_2_ and was determined during the first 100 s. The results were expressed as μmoL ascorbate oxidized mg^−1^ protein min^−1^.

#### 4.6.2. Glutathione Reductase (GR) Activity

GR (EC 1.6.4.2) activity was determined by following the oxidation of nicotinamide adenine dinucleotide phosphate (NADPH) at 340 nm (extinction coefficient 6.2 mM cm^−1^) as described by Rao [33]. The 1 mL assay mixture contained 100 mM potassium phosphate buffer (pH 7.8), 2 mM EDTA, 0.2 mM NADPH, and the plant extract. The assays were initiated by the addition of 0.5 mM GSSG at 25 °C. The activity was measured after monitoring NADPH oxidation for three absorbances was taken at 340 nm activity expressed as nmoL min^−1^ mg protein^−1^.

Activities of all studied antioxidative enzymes were converted to mg of protein. Total soluble protein was estimated according to the Bradford method [34] by using bovine serum albumin (BSA) as a standard. For spectrophotometric analyses, a spectrophotometer UNICO 2800 (United Products @ Instruments, Middlesex, NJ, USA) was used.

### 4.7. Statistical Analysis

All physiological and biochemical experiments were performed at three biological and three analytical replicates. The data were presented as the mean ± standard error (SEM). Statistically significant differences between the mean values were evaluated using analysis of variance (ANOVA), followed by the Tukey test (*p* < 0.05).

## 5. Conclusions

Thus, a genotypic specificity of the responses of wheat genotypes contrasting in drought sensitivity to endobacteria *B. subtilis* inoculation under drought was revealed. Which is associated with the ability of *B. subtilis* to regulate the components of the AsA–GSH complex as an effective antioxidant system. The result suggests the key role in the realization of *B. subtilis*-caused drought tolerance in plants of DS cv. Salavat Yulaev and DT cv. Ekada70 are AsA and GSH, respectively; which in both cases resulted in reduced oxidative damages of membranes and improved wheat plants’ growth under drought. The obtained results would be useful in developing drought-tolerant wheat cultivars associated with endobacteria *B. subtilis* in future breeding programs.

## Figures and Tables

**Table 1:** Effect of drought (12% PEG) on seed germination (three days after sowing) percentage and length of roots and shoots of four-days old *T. aestivum* L. (wheat) seedlings of drought-tolerant (DT) cv. Ekada70 and drought-susceptible (DS) cv. Salavat Yulaev, non-inoculated (C) and pre-inoculated with endobacterial *B. subtilis* strain 10–4 (BS). Drought exposure time—7 h. In the table presented is the average data of three repetitions (*n* = 50).

Growth Parameters		0% PEG (Non-Stressed)				12% PEG (Stressed)			
		Control (Non-Inoculated)		BS		Control (Non-Inoculated)		BS	
		DT	DS	DT	DS	DT	DS	DT	DS
Seed germination (%)		97 ± 1	96 ± 2	99 ± 1	97 ± 1	57 ± 2	39 ± 3	74 ± 2 *	42 ± 2
Lenght (cm)	Roots	2.40 ± 0.12	3.42 ± 0.09	2.59 ± 0.10 *	3.54 ± 0.14 *	2.16 ± 0.10	3.25 ± 0.09	2.41 ± 0.09 *	3.51 ± 0.09 *
	Shoots	3.05 ± 0.05	3.60 ± 0.07	3.26 ± 0.05 *	3.65 ± 0.06	2.44 ± 0.05	3.29 ± 0.07	3.24 ± 0.05 *	3.57 ± 0.06 *
FW (g)	Roots	0.60 ± 0.01	0.99 ± 0.02	0.65 ± 0.03	0.85 ± 0.09	0.53 ± 0.01	0.76 ± 0.02	0.56 ± 0.01	0.71 ± 0.06
	Shoots	0.66 ± 0.02	0.94 ± 0.05	0.63 ± 0.02	1.03 ± 0.02 *	0.38 ± 0.01	0.43 ± 0.04	0.41 ± 0.06	0.43 ± 0.008
DW (g)	Roots	0.065 ± 0.001	0.08 ± 0.009	0.059 ± 0.007	0.07 ± 0.01	0.06 ± 0.003	0.17 ± 0.03	0.062 ± 0.02	0.16 ± 0.008
	Shoots	0.050 ± 0.002	0.08 ± 0.007	0.057 ± 0.003	0.09 ± 0.008	0.04 ± 0.007	0.09 ± 0.01	0.05 ± 0.006	0.09 ± 0.005

* indicates a significant difference between the means at the level of *p* ˂ 0.05.

## Data Availability

Not applicable.

## Abbreviations

APX	ascorbate peroxidase
AsA	ascorbate
DS	drought susceptible
DT	drought tolerant
GR	glutathione reductase
GSH	glutathione
GSSG	glutathione disulfide (oxidized glutathione)
H_2_O_2_	hydrogen peroxide
MDA	malondialdehyde
PEG	polyethylene glycol
PGPB	plant growth-promoting bacteria
ROS	reactive oxygen species
